# Preliminary Clinical Outcomes of an Alternating-Current-Assisted Implant Surface Decontamination Protocol: A Prospective Case Series with 12-Month Follow-Up

**DOI:** 10.3390/bioengineering13090996

**Published:** 2026-08-27

**Authors:** Dorian Kostandini, Antonella Mattei, Giovanni Falisi, Arianna Viarchi, Davide Gerardi, Angelo Bruni, Edra Qorri, Lia A. Hoz-Rodriguez, Eduardo Basañes Rivera, Sofia Rastelli, Roberto Gatto, Marco Severino, Gianluca Botticelli

**Affiliations:** 1 Faculty of Dental Medicine, Department of Dentistry, Western Balkans University, 1001 Tirana, Albania; dorian.kostandini@wbu.edu.al; 2 Department of Life, Health and Environmental Sciences, University of L’Aquila, 67100 L’Aquila, Italy; antonella.mattei@univaq.it (A.M.); giovanni.falisi@univaq.it (G.F.); davide.gerardi@graduate.univaq.it (D.G.); sofia.rastelli@graduate.univaq.it (S.R.); roberto.gatto@univaq.it (R.G.); gianluca.botticelli@univaq.it (G.B.); 3 Department of Biotechnological and Applied Clinical Sciences, University of L’Aquila, 67100 L’Aquila, Italy; angelo.bruni@graduate.univaq.it; 4 Department of Dentistry, Faculty of Medical Sciences, Albanian University, 1001 Tirana, Albania; e.qorri@albanianuniversity.edu.al; 5 Periodontal Biology and Mineralized Tissue Laboratory, Postgraduate Studies and Research Division, School of Dentistry, National Autonomous University of México, Mexico City 04510, Mexico; liahoz@fo.odonto.unam.mx; 6 Department of Prosthodontics, Division of Graduate Studies, Autonomous University of Queretaro, Santiago de Querétaro 76010, Querétaro, Mexico; dresbasanez@yahoo.com; 7 School of Medicine, Department of Medicine, Odontostomatological University of Perugia, S. Andrea delle Fratte, 06132 Perugia, Italy; marco.severino@unipg.it

**Keywords:** oral biofilm, peri-implantitis, implant surface decontamination, mechanical debridement, alternating-current cleaning device

## Abstract

Background: Treatment of peri-implantitis remains challenging because biofilm removal from microstructured implant surfaces is often incomplete. The present study is an uncontrolled, prospective feasibility case series involving six patients (six implants), not a controlled clinical trial, in which short-term clinical outcomes following use of a multimodal protocol to decontaminate the titanium implant surface were obtained; consequently, the independent contribution of the Ximplant^®^ device to the observed outcomes cannot be determined. The protocol comprised surgical access, mechanical debridement, chemical conditioning, systemic administration of medication, and cleaning with an alternating-current device (Ximplant^®^; LED, S.p.A., Aprilia, Italy). Methods: Six adults with peri-implantitis, each contributing one implant, were consecutively treated using the multimodal protocol. Probing depth (PD), bleeding on probing (BOP), plaque index (PI), and suppuration (SU) were determined at baseline, 3 weeks, 6 months, and 12 months. Longitudinal changes were analyzed using repeated-measures analysis of variance and non-parametric tests for categorical variables. Results: Mean total PD decreased from 4.56 ± 0.45 mm at baseline to 3.03 ± 0.44 mm at the 3-week follow-up and remained stable at the 6-month follow-up (3.08 ± 0.35 mm) and at 12-month follow-up (3.19 ± 0.41 mm; *p* < 0.0001). BOP and SU were absent in all implants at each follow-up visit. PI improved markedly, although the buccal aspect of one implant was PI-positive at the 3-week and the 12-month follow-up. Conclusions: The multimodal protocol used in this study was associated with rapid and sustained clinical improvement over the 12-month follow-up period. A study limitation is that because the individual parts of the protocol were applied concurrently, the independent contribution of any of them to the outcomes determined could not be isolated.

## 1. Introduction

Dental implants have reported survival rates exceeding 90%; however, long-term success depends on factors related to the patient, implant, surgical site, and surrounding hard and soft tissues. Relevant determinants include age, sex, bone quality, implant location, timing of placement, and implant dimensions [1]. Despite favorable survival rates, the physicochemical characteristics and microtopography of titanium implant surfaces can facilitate bacterial adhesion and microbial colonization, potentially initiating inflammation in peri-implant tissues.

Peri-implant diseases may progress rapidly and can cause substantial tissue destruction; therefore, accurate and timely diagnosis is essential [2]. These conditions are commonly classified as peri-implant mucositis or peri-implantitis. Peri-implant mucositis is a biofilm-induced inflammatory condition confined to the soft tissues surrounding an implant. It is clinically characterized by bleeding on probing, with or without erythema, swelling, or suppuration, and without bone loss beyond initial physiological remodeling [3,4,5].

Peri-implantitis is a biofilm-associated inflammatory condition involving both the soft tissues and the supporting bone around an osseointegrated implant [6]. Clinical findings include bleeding on probing and/or suppuration, increased probing depth and/or mucosal recession, and radiographic evidence of progressive bone loss compared with previous examinations.

Accurate diagnosis of both peri-implant mucositis and peri-implantitis is a prerequisite for appropriate treatment planning. Initial management generally includes mechanical disruption and removal of the biofilm from the implant surface [7]. However, mechanical debridement alone may be insufficient because access is limited by the morphology of peri-implant bone defects, implant threads, and microstructured surfaces [8,9].

Consequently, several adjunctive strategies have been investigated to enhance implant surface decontamination, including ultrasonic instrumentation, air polishing, laser-based approaches, electrolytic cleaning, and electrical stimulation. Electrolytic cleaning protocols using potassium iodide and sodium formate have been evaluated [10], while electric-field approaches that do not require an electrolytic cleaning solution have also attracted interest. Experimental studies suggest that electrical stimulation may weaken microbial adhesion and reduce biofilm viability on implant materials [11,12]. Additional approaches include ultrasonic instrumentation and diode laser devices, as well as emerging strategies such as low-temperature plasma, erythritol air-polishing, pulsed electromagnetic field therapy, and artificial intelligence-guided nanoparticle delivery [13,14]. However, the available evidence remains heterogeneous, and the clinical evidence supporting individual adjunctive approaches is still limited. In particular, recent clinical evidence on Er laser and erythritol air-polishing has not demonstrated a clear additional clinical benefit over mechanical instrumentation alone [13]. Thus, despite promising experimental findings, evidence regarding electrical approaches remains limited, particularly concerning their clinical use within multimodal implant-surface decontamination protocols and the durability of clinical outcomes. The purpose of the present study was to report preliminary 12-month clinical outcomes of a standardized mechanical–surgical debridement protocol supplemented with use of an alternating-current device (Ximplant^®^; LED, S.p.A., Aprilia, Italy), for implant surface decontamination in patients with peri-implantitis. Because all components of the protocol were applied concurrently, this study was not designed to determine the independent efficacy of the alternating-current device.

## 2. Materials and Methods

### 2.1. Study Design and Ethical Considerations

This prospective case series included adults treated for peri-implantitis according to a standardized clinical protocol. The study was conducted in accordance with the Declaration of Helsinki. Written informed consent was obtained from all participants, and the clinical data were anonymized.

### 2.2. Population and Eligibility Criteria

Patients presenting to the Department of Odontostomatology at Western Balkans University, Albania, were screened clinically and radiographically. Given the exploratory case-series design, no formal sample-size calculation was performed. Of 10 patients screened for eligibility at the Department of Odontostomatology, 2 were excluded and 2 declined to participate. Six consecutive eligible participants were included (Table 1). Eligibility criteria were age ≥ 18 years; at least one peri-implant site with probing depth ≥ 5 mm; bleeding on probing and/or suppuration; radiographic bone loss ≥ 3 mm; and at least partially preserved bony walls.

The presence and extent of residual bony walls were assessed intraoperatively by visual and tactile examination of the peri-implant defect after flap elevation; the number of residual bone walls for each implant is reported in Table 1. Exclusion criteria were peri-implantitis treatment within the previous 6 months; untreated periodontitis; systemic conditions or medications that could impair healing; pregnancy; presence of a pacemaker; and implants considered hopeless or associated with non-retentive bone defects.

All participants received detailed information about the proposed treatment before enrollment and provided written informed consent.

For each participant, demographic and clinical data (age, sex, smoking status, systemic comorbidities, and oral hygiene habits) were collected, together with implant-related parameters, including implant position, diameter, and peri-implant defect morphology. Information regarding the specific implant system used, however, was not available for any of the six patients, as this information was not reported by the patients themselves.

### 2.3. Treatment Protocol

All procedures were performed by the same clinician to standardize treatment delivery.

#### 2.3.1. Perioperative Infection-Control Protocol

Professional peri-implant hygiene was performed before surgery to reduce the local microbial burden and stabilize the soft tissues. Patients were instructed to rinse with 0.2% chlorhexidine three times daily, beginning 1 week before surgery and continuing for 2 weeks postoperatively.

A systemic antibiotic regimen of amoxicillin/clavulanic acid (750/250 mg) twice daily for 8 days was prescribed, beginning 48 h before surgery. Ibuprofen 600 mg three times daily was prescribed for the first 48 postoperative hours.

#### 2.3.2. Surgical Access and Surface Decontamination

The surgical protocol was designed to provide direct access to the implant surface and peri-implant defect:

1. Removal of the prosthesis, when feasible, to facilitate access to the implant surface.
2. Administration of local anesthesia.
3. Intrasulcular incision and elevation of a full-thickness flap to expose the implant surface and defect morphology.
4. Removal of granulation tissue using titanium or carbon curettes (American Eagle Instruments, Barnhart 5/6–Langer 3/4, Missoula, MT, USA).
5. Sonic debridement using Sonic handpiece Proxeo (W&H (Dentalwerk Bürmoos), Bürmoos, Austria), sonic insert mod. SF1981.000 and PEEK tip (polyetheretherketone) SF1982.000 (Komet Italia, Verona, Italy) to disrupt adherent deposits while limiting surface damage.

#### 2.3.3. Ximplant^®^ Electrical Decontamination Phase

The Ximplant^®^ device delivered a high-frequency alternating electrical current. The following treatment settings were used:

- Citric acid application for 30–60 s.
- Irrigation with sterile saline solution.
- Ximplant^®^ electrical stimulation with the following parameters:

Frequency: 625 kHz

Peak-to-peak voltage: 260 Vpp

Power: 15 W

Delivery pattern: four 3 s active cycles separated by 2 s pauses

The device was operated according to the manufacturer’s protocol. This clinical study did not directly assess temperature changes, biofilm detachment, or the underlying biophysical mechanisms; therefore, no mechanistic conclusions can be drawn from the present data.

The implant surface and surgical field were then irrigated with sterile saline to remove residual chemical agents and detached material.

#### 2.3.4. Surgical Closure and Follow-Up

The flap was repositioned and sutured to obtain tension-free closure. Clinical and radiographic follow-up examinations were scheduled at 3 weeks, 6 months, and 12 months. PD, BOP, PI, and SU were recorded at each visit. Clinical measurements (PD, BOP, PI, SU) were obtained using a PREMIER periodontal probe (Premier Dental Products Company, Plymouth Meeting, PA, USA). Clinical measurements (PD, BOP, PI, and SU) were recorded by the same clinician who performed the surgical procedures; a separate, blinded examiner was not used, and formal inter- or intra-examiner calibration was not performed, which represents a limitation of this study. PD was measured to the nearest millimeter using a manual periodontal probe, applying light, standardized probing force, referenced to the prosthetic margin/implant shoulder.

## 3. Statistical Analysis

Because one implant was treated in each participant, the participant and implant constituted the same analytical unit. Continuous variables were summarized as mean ± standard deviation (SD), and categorical variables as frequencies and percentages. Total PD was defined as the mean of the six probing sites per implant; buccal PD and oral PD were defined as the mean of the three buccal or three oral sites per implant, respectively. The implant (n = 6) was the unit of analysis, and the 36 individual site-level measurements were not treated as independent observations. The distribution of PD values was assessed using the Shapiro–Wilk test. Because the data were non-normally distributed, PD values were logarithmically transformed before analysis. Changes in PD over time were evaluated using one-way repeated-measures analysis of variance, with time as the within-subject factor (four levels). Total PD change from baseline to 3 weeks was designated as the primary outcome; all other comparisons (buccal/oral PD, later timepoints, and categorical outcomes) were considered secondary and exploratory. Greenhouse–Geisser-corrected *p*-values are reported, and Bonferroni-adjusted pairwise comparisons were performed. Changes in BOP, PI, and SU over follow-up time were analyzed using Cochran’s Q test, followed by McNemar’s test for pairwise comparisons. No correction for multiplicity was applied across the multiple pairwise comparisons of categorical outcomes; given the small sample size, *p*-values close to the significance threshold should be interpreted with caution and considered together with the absolute frequencies. Given the very small sample size (n = 6), point estimates of effect size and their confidence intervals were not calculated, as these would convey a level of precision not supported by the data. Analyses were performed using Stata/BE 18.0 (StataCorp LLC, College Station, TX, USA). All tests were two-sided, and *p* < 0.05 was considered statistically significant. Given the small sample, all analyses were considered exploratory. Given the very small sample size, these results should be regarded as descriptive and hypothesis-generating rather than as confirmatory statistical inference.

## 4. Results

Six adults (four women and two men; age: 60.8 ± 8.1 years) with peri-implantitis were included. One osseointegrated implant per participant was treated, for a total of six implants supporting single crowns (4 participants) or fixed partial dentures (2 participants).

At baseline (T0), each implant had at least one site meeting the eligibility criteria for peri-implantitis. All implants were positive for BOP and PI and showed suppuration at one or more sites. Each implant was examined at six sites: mesiobuccal, mid-buccal, disto-buccal, mesio-oral, mid-oral, and disto-oral were obtained at baseline (T0), 3 weeks (T1), 6 months (T2), and 12 months (T3).

**Figure 1:**
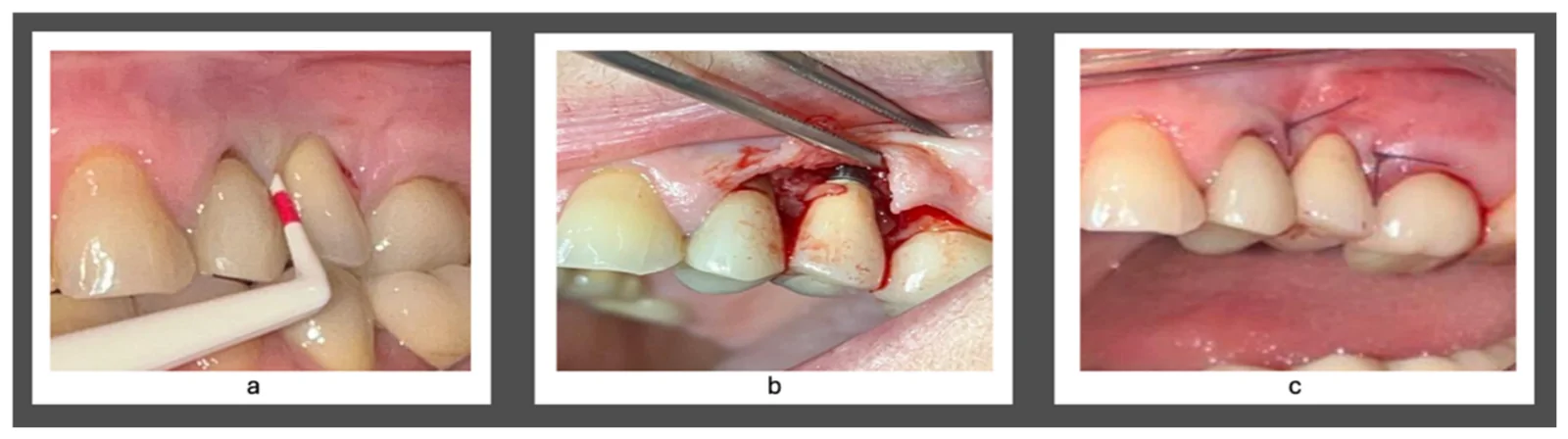
Treatment protocol: (**a**) baseline probing assessment at the buccal aspect; (**b**) elevation of a full-thickness flap and exposure of the peri-implant bone defect; and (**c**) interrupted sutures after completion of the Ximplant^®^-assisted decontamination protocol.

Baseline clinical assessment was followed by the multimodal treatment protocol (Figure 1).

**Figure 2:**
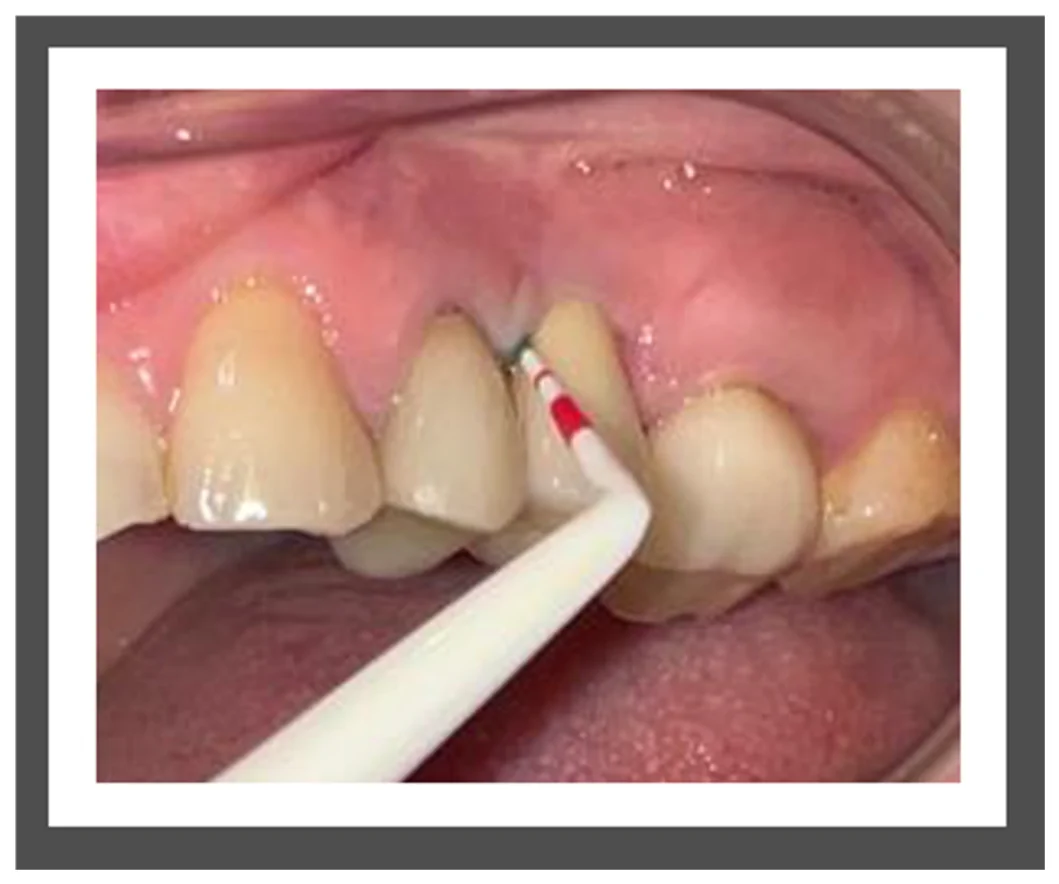
Clinical probing assessment at the buccal aspect 12 months after treatment.

**Figure 3:**
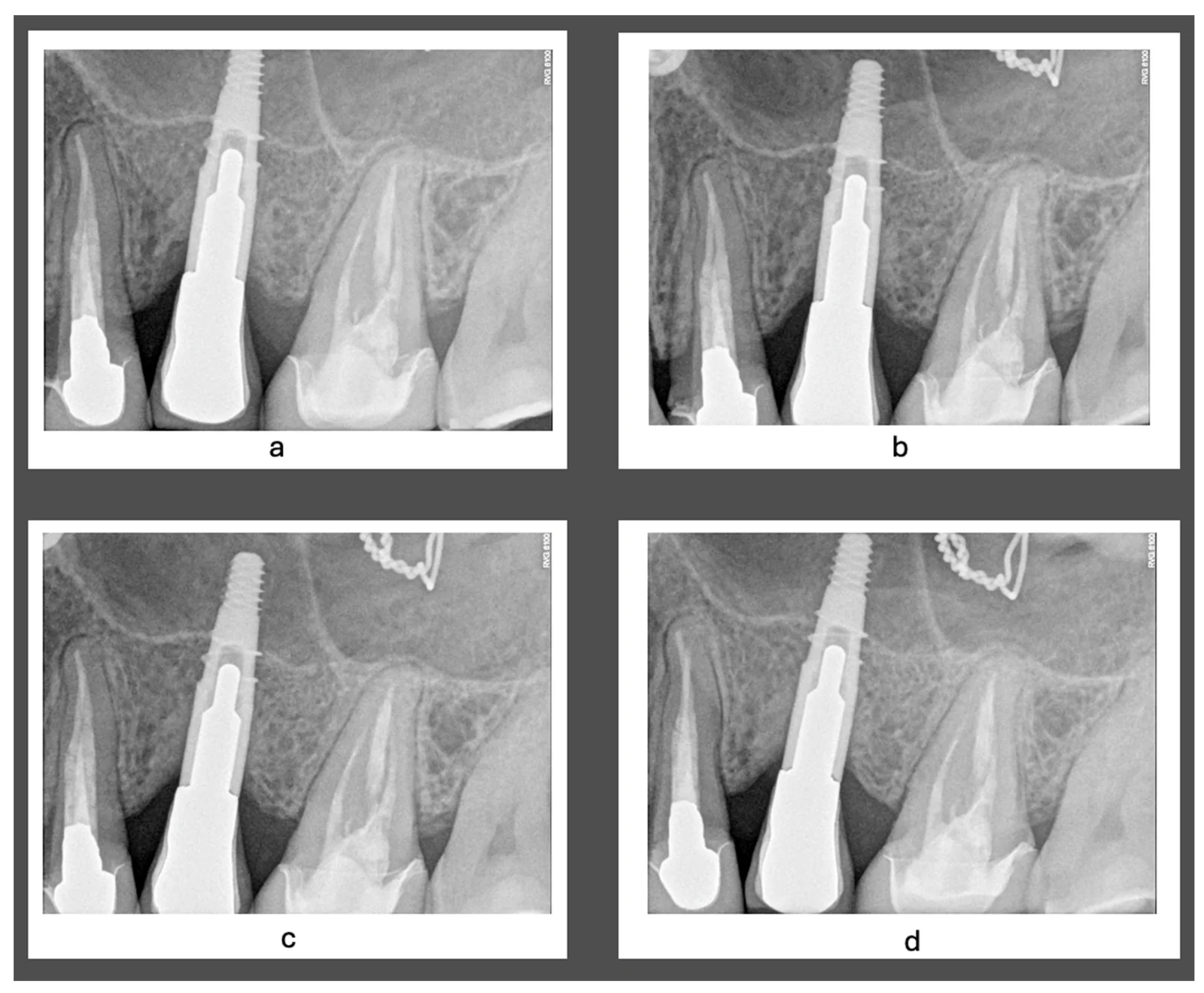
Intraoral radiographs acquired using an RVG 6100 digital sensor (Carestream Dental; resolution > 20 lp/mm) at baseline (**a**), 3 weeks (**b**), 6 months (**c**), and 12 months (**d**).

**Figure 4:**
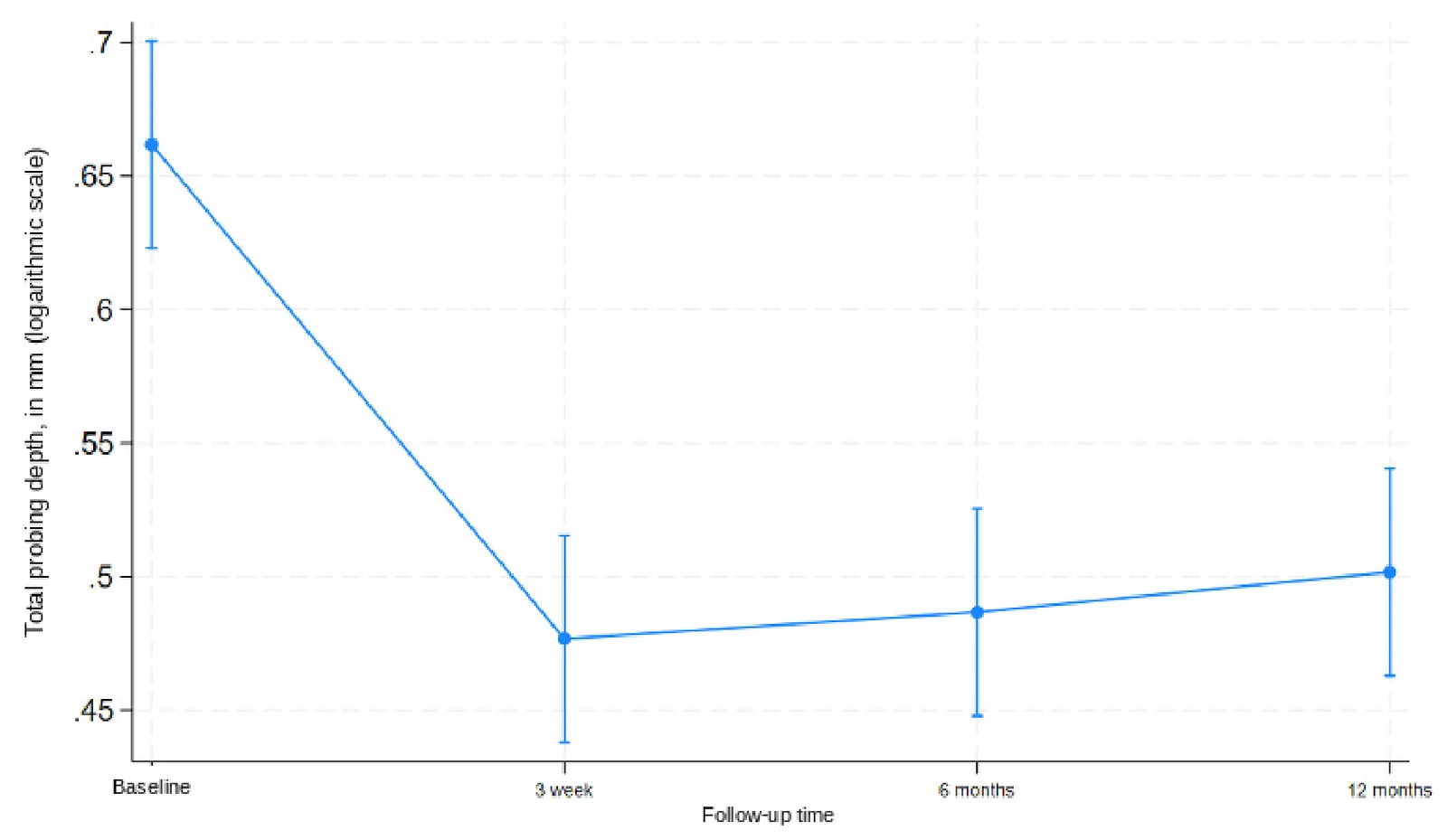
Variation in logarithmically transformed total probing depth with follow-up time.

**Figure 5:**
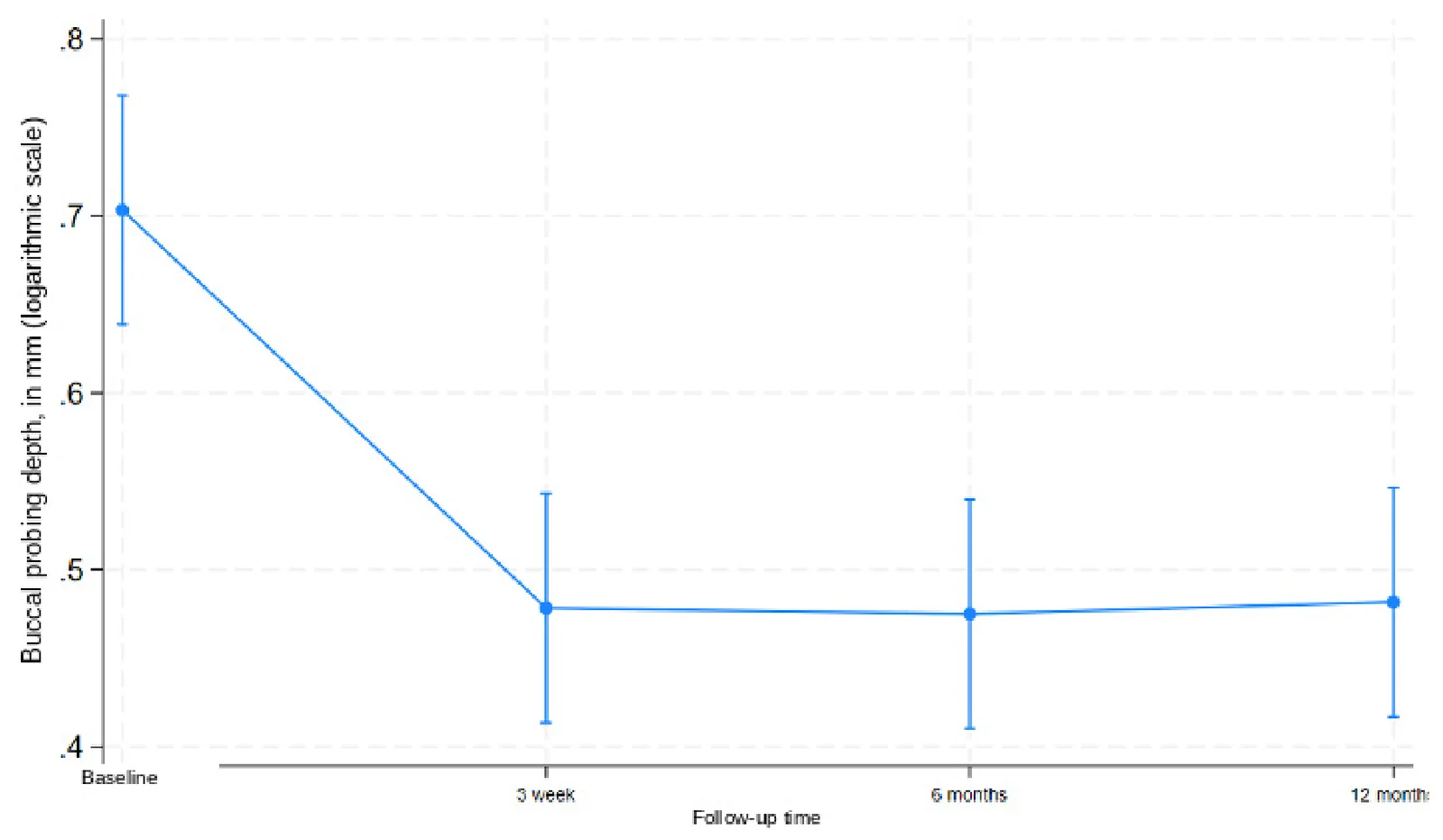
Variation in logarithmically transformed buccal probing depth with follow-up time.

**Figure 6:**
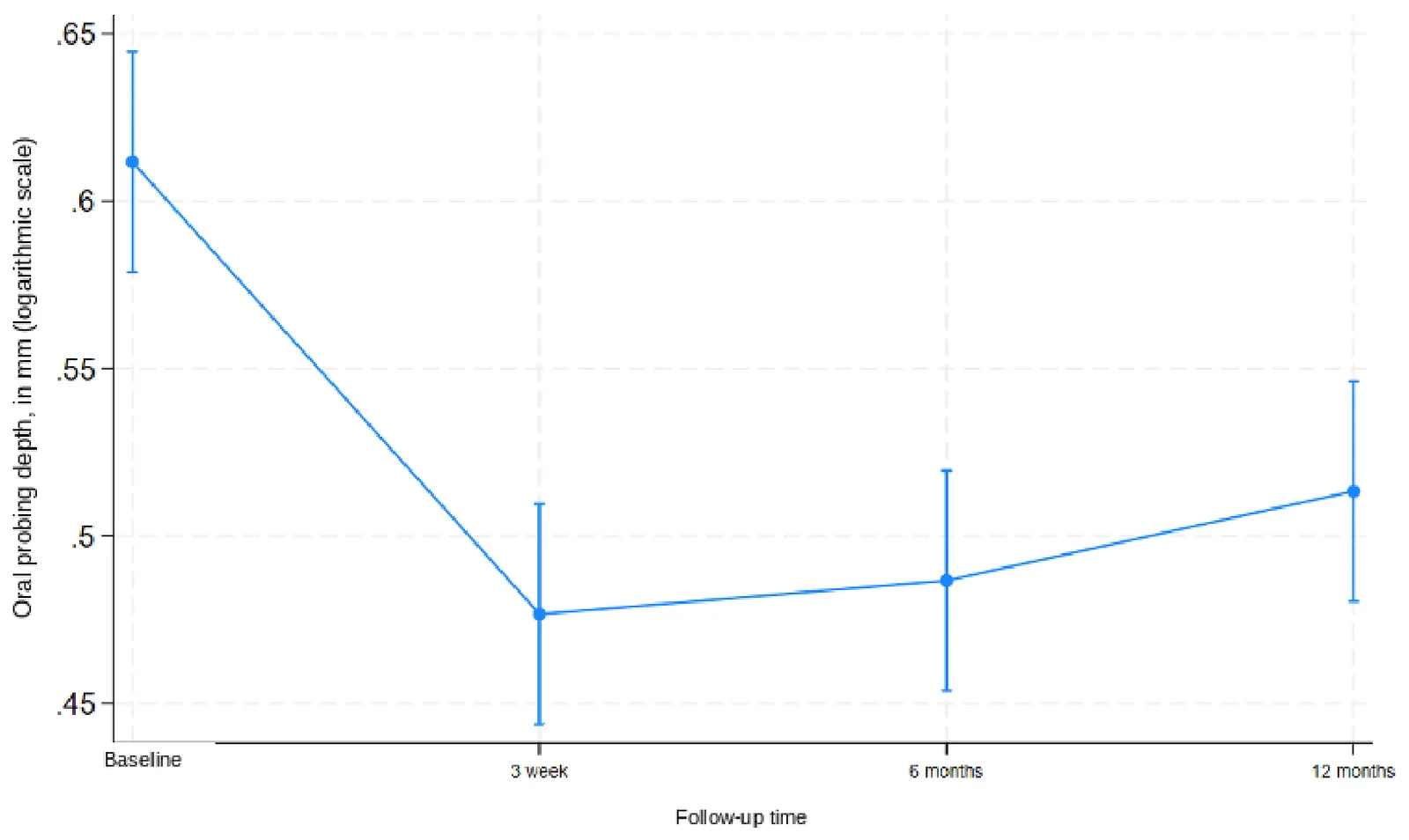
Variation in logarithmically transformed oral probing depth with follow-up time.

Follow-up clinical and radiographic assessments are illustrated in Figure 2 and Figure 3. Overall, PD, BOP, PI, and SU improved relative to baseline. The clinical outcomes over the follow-up time and statistical analyses of them are presented in Table 2, Table 3, Table 4 and Table 5 and Figure 4, Figure 5 and Figure 6.

### Clinical Outcomes

For each implant, total PD was calculated as the mean of all six probing sites (mesio-buccal, mid-buccal, disto-buccal, mesio-oral, mid-oral, and disto-oral); buccal PD and oral PD were calculated as the mean of the three respective buccal or oral sites at each assessment time. Individual-level PD, BOP, PI, and SU values at each follow-up visit for every participant/implant are provided in the Supplementary Materials (File S1).

A statistically significant reduction in PD was observed over time for total, buccal, and oral measurements (all *p* < 0.0001). Mean total PD decreased from 4.56 ± 0.45 mm at baseline to 3.03 ± 0.44 mm at the 3-week follow-up and remained similar at the 6-month follow-up (3.08 ± 0.35 mm) and 12-month follow-up (3.19 ± 0.41 mm). Similar trends were seen in the buccal and oral PD results (Table 2).

There were significant reductions in total, buccal, and oral PD between baseline and each follow-up time (each *p* < 0.001; Table 3. The largest reductions occurred in the 3-week follow-up, after which mean values remained stable up to the 12-month follow-up.

Variations in logarithmically transformed PDs and their associated 95% confidence intervals with follow-up time are presented in Figure 4. Each PD measure dropped markedly between the baseline and the 3-week follow-up. After that, total PD rose very slightly, buccal PD was practically unchanged, and oral PD rose very slightly.

Buccal PD showed a similar marked decrease at 3 weeks, with little change at the 6-month follow-up and the 12-month follow-up (Figure 5).

Oral PD also decreased markedly at the 3-week follow-up. A small increase was observed between the 6-month follow-up and the 12-month follow-up, although values remained lower than at baseline (Figure 6).

With three exceptions, BOP, PI, and SU improved significantly over the total follow-up time (Table 4). The exceptions were that one implant was totally PI-positive at the 12-week follow-up, one was PI-positive at the buccal aspect at the 3-week follow-up, and one was PI-positive at the buccal aspect at the 12-week follow-up.

With three exceptions, significant changes in BOP, PI, and SU occurred with pairs of follow-up times (Table 5). The exceptions were for total PI (T0 vs. T3), buccal PI (T0 vs. T1), and buccal PI (T0 vs. T3).

## 5. Discussion

Peri-implantitis remains challenging to treat because implant surface characteristics, thread geometry, and peri-implant defect morphology [15] may limit effective biofilm removal by mechanical debridement alone [5,6,16,17]. Consequently, adjunctive approaches have been introduced to improve implant-surface decontamination, including ultrasonic instruments, air-polishing devices, and laser-based procedures [18,19]. Electrical-current-based decontamination represents a more recent approach. Experimental evidence suggests that electrical stimulation may interfere with bacterial adhesion and viability on implant materials [11,12,20,21]. Nevertheless, no single surface-decontamination approach has demonstrated consistent superiority [16,22,23,24,25]. Against this background, the present prospective case series evaluated preliminary 12-month clinical outcomes following a multimodal protocol combining surgical access, mechanical debridement, chemical conditioning, systemic medication, and alternating-current-assisted implant-surface cleaning.

The present findings showed a marked early improvement in the evaluated clinical parameters. Mean total PD decreased from 4.56 ± 0.45 mm at baseline to 3.03 ± 0.44 mm at 3 weeks and remained relatively stable at 6 and 12 months, with similar trends for buccal and oral PD. The early reduction in PD may reflect the combined effects of direct surgical access, removal of granulation tissue and adherent deposits, mechanical debridement, and chemical conditioning. BOP and SU were absent in all implants from 3 weeks through 12 months, indicating sustained control of clinically detectable peri-implant inflammation. PI also improved substantially, although isolated buccal plaque positivity was observed at 3 weeks and 12 months. This residual plaque accumulation emphasizes the importance of continued plaque control during supportive peri-implant care. Because all treatment components were applied concurrently, the observed improvements must be attributed to the complete multimodal protocol rather than to the alternating-current device alone.

Previous studies have investigated different adjunctive approaches for peri-implant decontamination. Mettraux et al. [26] reported a reduction in BOP following repeated non-surgical mechanical therapy combined with adjunctive diode-laser application. In the present study, BOP and SU were absent at all follow-up visits, while PD showed a marked reduction at 3 weeks and remained relatively stable through 12 months. Experimental studies evaluating electrical stimulation have also reported effects on bacterial adhesion and viability on implant materials [11,12,20,21]. However, these experimental findings cannot be directly compared with the clinical outcomes observed in the present case series. Therefore, the present results should be interpreted as preliminary clinical outcomes of the complete multimodal protocol rather than as evidence of the independent efficacy of the alternating-current cleaning device.

The main novelty of the present study is the clinical evaluation of a comprehensive multimodal protocol incorporating surgical access, mechanical debridement, chemical conditioning, systemic medication, and alternating-current-assisted implant-surface cleaning, with clinical outcomes monitored over 12 months. However, several limitations should be acknowledged. The very small sample size and the single-center, single-operator setting limit the generalizability of the findings. In addition, information about patient and implant characteristics that may affect treatment outcome, such as diabetes and design, respectively, was not known. Microbiological, patient-reported, and quantitative radiographic outcomes were also not assessed. The radiographs are provided for qualitative illustration only, as projection geometry, angulation, exposure, and magnification were not standardized across visits; therefore, they should not be used to infer bone stability or regeneration. Most importantly, the simultaneous application of all treatment components prevents determination of their independent contributions to the observed outcomes and, specifically, does not allow the efficacy of the alternating-current device to be established. Furthermore, the device was evaluated using only one set of operating conditions, including a power setting of 15 W. Adherence to the prescribed chlorhexidine and antibiotic regimens, as well as maintenance care and any deviations from postoperative instructions between visits, was not objectively monitored, which represents an additional limitation of this study. Future multi-arm randomized controlled trials with a larger samples and longer follow-up should evaluate the complete protocol and its individual components, investigate the influence of different operating conditions, including power levels between 15 and 25 W, incorporate remote or continuous monitoring of oral hygiene behavior, such as smartphone-based telemonitoring of toothbrushing [27], to objectively capture post-treatment adherence, and obtain additional outcomes, such as implant-surface characteristics and osseointegration status after decontamination.

## 6. Conclusions

In this prospective case series of six patients, a combined mechanical–surgical, chemical, pharmacological, and alternating-current-assisted protocol was associated with an early reduction in PD and resolution of BOP and suppuration that was maintained through 12 months. PI also improved substantially but was not absent in every implant at all follow-up visits. Because the study was uncontrolled and the electrical device was used within a multimodal protocol, neither efficacy nor the independent contribution of Ximplant^®^ can be established. Randomized controlled clinical trials are needed to determine comparative effectiveness, safety, and indications.

## Figures and Tables

**Table 1:** Patient and implant characteristics.

Patient	Age (Year)	Sex	Smoking Yes/No	Comorbidities Yes/No	Toothbrushing Frequency/Day	Mouthwash Yes/No	Implant Position	Implant System	Implant Diameter (mm)	Defect Morphology
01	65	F	YES	NO	2 times	NO	2.5	NR *	3.5	4 walls defect
02	69	F	NO	NO	3 times	NO	1.3	NR *	3.8	2 walls defect
03	63	F	NO	NO	1 time	YES	3.3	NR *	3.8	4 walls defect
04	70	F	YES	NO	3 times	YES	3.5	NR *	4.0	2 walls defect
05	60.8	M	NO	NO	2 times	NO	2.6	NR *	4.0	4 walls defect
06	68.9	M	NO	NO	2 times	YES	4.7	NR *	4.5	3 walls defect

* NR (not reported). The patient did not report the implant system.

**Table 2:** Probing depths (PD) (in mm) results.

	Baseline	3-Week Follow-Up	6-Month Follow-Up	12-Month Follow-Up	*p*-Value *
Total PD, mean ± SD	4.56 ± 0.45	3.03 ± 0.44	3.08 ± 0.35	3.19 ± 0.41	**<0.0001**
Buccal PD, mean ± SD	5.05 ± 0.44	3.06 ± 0.61	3.05 ± 0.68	3.11 ± 0.81	**<0.0001**
Oral PD, mean ± SD	4.11 ± 0.58	3.00 ± 0.37	3.06 ± 0.13	3.27 ± 0.25	**<0.0001**

* One-way repeated-measures ANOVA; Greenhouse–Geisser-corrected *p*-value.

**Table 3:** Bonferroni-adjusted pairwise comparisons of probing depth.

	Baseline vs. 3-Week Follow-Up	Baseline vs. 6-Month Follow-Up	Baseline vs. 12-Month Follow-Up
Total PD	<0.001	<0.001	<0.001
Buccal PD	<0.001	<0.001	<0.001
Oral PD	<0.001	<0.001	<0.001

**Table 4:** Clinical characteristics according to follow-up time.

	Baseline	3-Week Follow-Up	6-Month Follow-Up	12-Month Follow-Up	*p*-Value *
Total BOP, n (%)					**0.0004**
No	0 (0.00)	6 (100.00)	6 (100.00)	6 (100.00)	
Yes	6 (100.00)	0 (0.00)	0 (0.00)	0 (0.00)	
Buccal BOP, n (%)					**0.0004**
No	0 (0.00)	6 (100.00)	6 (100.00)	6 (100.00)	
Yes	6 (100.00)	0 (0.00)	0 (0.00)	0 (0.00)	
Oral BOP, n (%)					**0.0004**
No	0 (0.00)	6 (100.00)	6 (100.00)	6 (100.00)	
Yes	6 (100.00)	0 (0.00)	0 (0.00)	0 (0.00)	
Total PI, n (%)					**0.0013**
No	0 (0.00)	6 (100.00)	6 (100.00)	5 (83.33)	
Yes	6 (100.00)	0 (0.00)	0 (0.00)	1 (16.67)	
Buccal PI, n (%)					**0.0042**
No	0 (0.00)	5 (83.33)	6 (100.00)	5 (83.33)	
Yes	6 (100.00)	1 (16.67)	0 (0.00)	1 (16.67)	
Oral PI, n (%)					**0.0004**
No	0 (0.00)	6 (100.00)	6 (100.00)	6 (100.00)	
Yes	6 (100.00)	0 (0.00)	0 (0.00)	0 (0.00)	
Total SU, n (%)					**0.0004**
No	0 (0.00)	6 (100.00)	6 (100.00)	6 (100.00)	
Yes	6 (100.00)	0 (0.00)	0 (0.00)	0 (0.00)	
Buccal SU, n (%)					**0.0004**
No	0 (0.00)	6 (100.00)	6 (100.00)	6 (100.00)	
Yes	6 (100.00)	0 (0.00)	0 (0.00)	0 (0.00)	
Oral SU, n (%)					**0.0018**
No	1 (16.67)	6 (100.00)	6 (100.00)	6 (100.00)	
Yes	5(83.33)	0 (0.00)	0 (0.00)	0 (0.00)	

* Cochran’s Q test.

**Table 5:** Pairwise comparisons of categorical clinical outcomes using McNemar’s test.

	T0 vs. T1	T1 vs. T2	T2 vs. T3	T0 vs. T3
Total BOP	**0.0312**	1.00	1.00	**0.0312**
Buccal BOP	**0.0312**	1.00	1.00	**0.0312**
Oral BOP	**0.0312**	1.00	1.00	**0.0312**
Total PI	**0.0312**	1.00	1.00	0.0625
Buccal PI	0.0625	1.00	1.00	0.0625
Oral PI	**0.0312**	1.00	1.00	**0.0312**
Total SU	**0.0312**	1.00	1.00	**0.0312**
Buccal SU	**0.0312**	1.00	1.00	**0.0312**
Oral SU	**0.0312**	1.00	1.00	**0.0312**

Values are *p*-values from McNemar’s test. T0: baseline; T1: 3 weeks; T2: 6 months; T3: 12 months.

## Data Availability

All data generated or analyzed during this study are included in this article. Additional de-identified data may be made available by the corresponding author upon reasonable request, subject to ethical and legal restrictions.

## Supplementary Materials

The following supporting information can be downloaded at: https://www.mdpi.com/article/10.3390/bioengineering13090996/s1, File S1: Individual-level probing depth (PD), bleeding on probing (BOP), plaque index (PI), and suppuration (SU) values at each follow-up visit for every participant/implant.

## Abbreviations

The following abbreviations are used in this manuscript:

BOP	bleeding on probing
CI	confidence interval
PD	probing depth
PI	plaque index
SU	suppuration
