# Supplementary material for: Preliminary Clinical Outcomes of an Alternating-Current-Assisted Implant Surface Decontamination Protocol: A Prospective Case Series with 12-Month Follow-Up
Source: Bioengineering (Basel). 2026 Aug 27;13(9):996. doi: 10.3390/bioengineering13090996 (PMC13603563; doi:10.3390/bioengineering13090996)
Supplement: Supplementary file 1 [file bioengineering-13-00996-s001.zip › bioengineering-4508064-supplementary.pdf]

bioengineering

MDPI

*Supplementary Materials*

# Preliminary Clinical Outcomes of an Alternating-Current-Assisted Implant Surface Decontamination Protocol: A Prospective Case Series with 12-Month Follow-Up

**Dorian Kostandini [1], Antonella Mattei [2], Giovanni Falisi [2], Arianna Viarchi [2,*], Davide Gerardi [2], Angelo Bruni [3], Edra Qorri [4], Lia A. Hoz-Rodriguez [5], Eduardo Basañes Rivera [6], Sofia Rastelli [2], Roberto Gatto [2], Marco Severino [7] and Gianluca Botticelli [2]**

[1] Faculty of Dental Medicine, Department of Dentistry, Western Balkans University, 1001 Tirana, Albania; dorian.kostandini@wbu.edu.al
[2] Department of Life, Health and Environmental Sciences, University of L'Aquila, 67100 L'Aquila, Italy; antonella.mattei@univaq.it (A.M.); giovanni.falisi@univaq.it (G.F.); davide.gerardi@graduate.univaq.it (D.G.); sofia.rastelli@graduate.univaq.it (S.R.); roberto.gatto@univaq.it (R.G.); gianluca.botticelli@univaq.it (G.B.)
[3] Department of Biotechnological and Applied Clinical Sciences, University of L'Aquila, 67100 L'Aquila, Italy; angelo.bruni@graduate.univaq.it
[4] Department of Dentistry, Faculty of Medical Sciences, Albanian University, 1001 Tirana, Albania; e.qorri@albanianuniversity.edu.al
[5] Periodontal Biology and Mineralized Tissue Laboratory, Postgraduate Studies and Research Division, School of Dentistry, National Autonomous University of México, Mexico City 04510, Mexico; liahoz@fo.odonto.unam.mx
[6] Department of Prosthodontics, Division of Graduate Studies, Autonomous University of Queretaro, Santiago de Querétaro 76010, Querétaro, Mexico; dresbasanez@yahoo.com
[7] School of Medicine, Department of Medicine, Odontostomatological University of Perugia, S. Andrea delle Fratte, 06132 Perugia, Italy; marco.severino@unipg.it
* Correspondence: arianna.viarchi@graduate.univaq.it

**Table S1. Dataset case 1:** Data from on-site surveys conducted at baseline, 3 weeks, 6 months and 12 months.

| Case 1 | Baseline | | | | 3 weeks | | | | 6 months | | | | 12 months | | | |
|---|---|---|---|---|---|---|---|---|---|---|---|---|---|---|---|---|
| Site | PD | BOP | PI | Su | PD | BOP | PI | SU | PD | BOP | PI | SU | PD | BOP | PI | SU |
| MV | 6 | 1 | 1 | 1 | 3 | 0 | 0 | 0 | 3 | 0 | 0 | 0 | 3 | 0 | 0 | 0 |
| V | 4 | 1 | 1 | 1 | 2 | 0 | 0 | 0 | 3 | 0 | 0 | 0 | 3 | 0 | 0 | 0 |
| DV | 5 | 1 | 1 | 1 | 3 | 0 | 0 | 0 | 3 | 0 | 0 | 0 | 3 | 0 | 0 | 0 |
| MO | 4 | 1 | 1 | 1 | 3 | 0 | 0 | 0 | 2 | 0 | 0 | 0 | 3 | 0 | 0 | 0 |
| O | 4 | 1 | 1 | 1 | 3 | 0 | 0 | 0 | 3 | 0 | 0 | 0 | 3 | 0 | 0 | 0 |
| DO | 5 | 1 | 1 | 1 | 2 | 0 | 0 | 0 | 4 | 1 | 1 | 0 | 3 | 0 | 0 | 0 |

**Table S2. Dataset case 2:** Data from on-site surveys conducted at baseline, 3 weeks, 6 months and 12 months.

| Case 2 | Baseline | | | | 3 weeks | | | | 6 months | | | | 12 months | | | |
|---|---|---|---|---|---|---|---|---|---|---|---|---|---|---|---|---|
| Site | PD | BOP | PI | Su | PD | BOP | PI | SU | PD | BOP | PI | SU | PD | BOP | PI | SU |
| MV | 5 | 1 | 1 | 1 | 2 | 0 | 0 | 0 | 2 | 0 | 0 | 0 | 2 | 0 | 0 | 0 |
| V | 3 | 1 | 1 | 1 | 2 | 0 | 0 | 0 | 2 | 0 | 0 | 0 | 2 | 0 | 0 | 0 |
| DV | 8 | 1 | 1 | 1 | 4 | 0 | 0 | 0 | 3 | 0 | 0 | 0 | 2 | 0 | 0 | 0 |
| MO | 3 | 1 | 1 | 1 | 3 | 0 | 0 | 0 | 2 | 0 | 0 | 0 | 3 | 0 | 0 | 0 |
| O | 3 | 1 | 1 | 1 | 3 | 0 | 0 | 0 | 3 | 0 | 0 | 0 | 4 | 0 | 0 | 0 |
| DO | 6 | 1 | 1 | 1 | 3 | 0 | 0 | 0 | 4 | 1 | 1 | 0 | 4 | 1 | 1 | 0 |

**Table S3. Dataset case 3:** Data from on-site surveys conducted at baseline, 3 weeks, 6 months and 12 months.

| Case 3 | Baseline | | | | 3 weeks | | | | 6 months | | | | 12 months | | | |
|---|---|---|---|---|---|---|---|---|---|---|---|---|---|---|---|---|
| Site | PD | BOP | PI | Su | PD | BOP | PI | SU | PD | BOP | PI | SU | PD | BOP | PI | SU |
| MV | 5 | 1 | 1 | 1 | 3 | 0 | 0 | 0 | 3 | 0 | 0 | 0 | 4 | 1 | 1 | 0 |
| V | 5 | 1 | 1 | 1 | 3 | 0 | 0 | 0 | 3 | 0 | 0 | 0 | 4 | 0 | 1 | 0 |
| DV | 5 | 1 | 1 | 1 | 2 | 0 | 0 | 0 | 3 | 0 | 0 | 0 | 4 | 0 | 1 | 0 |
| MO | 4 | 1 | 1 | 1 | 3 | 0 | 0 | 0 | 2 | 0 | 0 | 0 | 3 | 0 | 0 | 0 |
| O | 4 | 1 | 1 | 1 | 3 | 0 | 0 | 0 | 3 | 0 | 0 | 0 | 3 | 0 | 0 | 0 |
| DO | 5 | 1 | 1 | 1 | 3 | 0 | 0 | 0 | 3 | 0 | 0 | 0 | 4 | 1 | 1 | 0 |

**Table S4. Dataset Pz 4:** Data from on-site surveys conducted at baseline, 3 weeks, 6 months and 12 months.

| Case 4 | Baseline | | | | 3 weeks | | | | 6 months | | | | 12 months | | | |
|---|---|---|---|---|---|---|---|---|---|---|---|---|---|---|---|---|
| Site | PD | BOP | PI | Su | PD | BOP | PI | SU | PD | BOP | PI | SU | PD | BOP | PI | SU |
| MV | 6 | 1 | 1 | 1 | 4 | 0 | 0 | 0 | 4 | 0 | 0 | 0 | 3 | 0 | 0 | 0 |
| V | 4 | 1 | 1 | 1 | 3 | 0 | 0 | 0 | 3 | 0 | 0 | 0 | 3 | 0 | 0 | 0 |
| DV | 7 | 1 | 1 | 1 | 5 | 1 | 1 | 0 | 5 | 1 | 1 | 0 | 5 | 1 | 1 | 0 |
| MO | 5 | 1 | 1 | 1 | 3 | 0 | 0 | 0 | 2 | 0 | 0 | 0 | 3 | 0 | 0 | 0 |
| O | 4 | 1 | 1 | 1 | 3 | 0 | 0 | 0 | 3 | 0 | 0 | 0 | 3 | 0 | 0 | 0 |
| DO | 4 | 1 | 1 | 1 | 3 | 0 | 0 | 0 | 4 | 1 | 1 | 0 | 4 | 0 | 0 | 0 |

**Table S5. Dataset Pz 5:** Data from on-site surveys conducted at baseline, 3 weeks, 6 months and 12 months.

| Case 5 | Baseline | | | | 3 weeks | | | | 6 months | | | | 12 months | | | |
|---|---|---|---|---|---|---|---|---|---|---|---|---|---|---|---|---|
| Site | PD | BOP | PI | Su | PD | BOP | PI | SU | PD | BOP | PI | SU | PD | BOP | PI | SU |
| MV | 6 | 1 | 1 | 1 | 3 | 0 | 0 | 0 | 3 | 0 | 0 | 0 | 2 | 0 | 0 | 0 |
| V | 4 | 1 | 1 | 1 | 2 | 0 | 0 | 0 | 3 | 0 | 0 | 0 | 2 | 0 | 0 | 0 |
| DV | 3 | 1 | 1 | 1 | 3 | 0 | 0 | 0 | 3 | 0 | 0 | 0 | 3 | 0 | 0 | 0 |
| MO | 3 | 1 | 1 | 0 | 3 | 0 | 0 | 0 | 3 | 0 | 0 | 0 | 3 | 0 | 0 | 0 |
| O | 3 | 1 | 1 | 0 | 3 | 0 | 0 | 0 | 3 | 0 | 0 | 0 | 3 | 0 | 0 | 0 |
| DO | 3 | 1 | 1 | 0 | 2 | 0 | 0 | 0 | 3 | 0 | 0 | 0 | 3 | 0 | 0 | 0 |

**Table S6. Dataset Pz 6:** Data from on-site surveys conducted at baseline, 3 weeks, 6 months and 12 mont.

| ase 6 | Baseline | | | | 3 weeks | | | | 6 months | | | | 12 months | | | |
|---|---|---|---|---|---|---|---|---|---|---|---|---|---|---|---|---|
| **Site** | **PD** | **BOP** | **PI** | **Su** | **PD** | **BOP** | **PI** | **SU** | **PD** | **BOP** | **PI** | **SU** | **PD** | **BOP** | **PI** | **SU** |
| **MV** | 5 | 1 | 1 | 1 | 3 | 0 | 0 | 0 | 3 | 0 | 0 | 0 | 3 | 0 | 0 | 0 |
| **V** | 5 | 1 | 1 | 1 | 4 | 1 | 1 | 0 | 4 | 0 | 0 | 0 | 4 | 0 | 0 | 0 |
| **DV** | 5 | 1 | 1 | 1 | 4 | 1 | 1 | 0 | 4 | 0 | 0 | 0 | 4 | 0 | 0 | 0 |
| **MO** | 4 | 1 | 1 | 1 | 4 | 0 | 0 | 0 | 3 | 0 | 0 | 0 | 3 | 0 | 0 | 0 |
| **O** | 4 | 1 | 1 | 1 | 3 | 0 | 0 | 0 | 3 | 0 | 0 | 0 | 3 | 0 | 0 | 0 |
| **DO** | 5 | 1 | 1 | 1 | 4 | 0 | 0 | 0 | 4 | 1 | 1 | 0 | 4 | 0 | 0 | 0 |

*Table key*

**PD** - Probing Depth: MV (mesio-distal), V (vestibular), DV (disto-vestibular), MO (Mesio-oral) , O (Oral), DO (Distal- Oral).

**BOP** - Probing Bleeding: 0 value: no bleeding; Value 1: Positive bleeding after insertion of the probe.

**PI** - Plaque Index: Value 0, no plaque accumulation; Value 1, detection of plaque accumulation using the periodontal probe.

**SU** - Suppuration: Value 0, no suppuration; Value 1, presence of suppuration.
